# Targeting the Mild-Hypoxia Driving Force for Metabolic and Muscle Transcriptional Reprogramming of Gilthead Sea Bream (*Sparus aurata*) Juveniles

**DOI:** 10.3390/biology10050416

**Published:** 2021-05-08

**Authors:** Fernando Naya-Català, Juan A. Martos-Sitcha, Verónica de las Heras, Paula Simó-Mirabet, Josep À. Calduch-Giner, Jaume Pérez-Sánchez

**Affiliations:** 1 Nutrigenomics and Fish Growth Endocrinology Group, Institute of Aquaculture Torre de la Sal, CSIC, 12595 Ribera de Cabanes, Spain; fernando.naya@iats.csic.es (F.N.-C.); juanantonio.sitcha@uca.es (J.A.M.-S.); veronica.delasher@alum.uca.es (V.d.l.H.); paula.simo@csic.es (P.S.-M.); j.calduch@csic.es (J.À.C.-G.); 2 Department of Biology, Faculty of Marine and Environmental Sciences, Instituto Universitario de Investigación Marina (INMAR), Campus de Excelencia Internacional del Mar (CEI-MAR), University of Cádiz, 11519 Cádiz, Spain

**Keywords:** hypoxia, hypo-metabolic state, growth, swimming performance, metabolic landmarks, muscle transcriptome, glycolysis, lipid metabolism, protein turnover, gilthead sea bream

## Abstract

**Simple Summary:**

Reduced oxygen availability generates a number of adaptive features across all the animal kingdom, and the goal of this study was targeting the mild-hypoxia driving force for metabolic and muscle transcriptional reprogramming of gilthead sea bream juveniles. Attention was focused on blood metabolic and muscle transcriptomic landmarks before and after exhaustive exercise. Our results after mild-hypoxia conditioning highlighted an increased contribution of lipid metabolism to whole energy supply to preserve the aerobic energy production, a better swimming performance regardless of changes in feed intake, as well as reduced protein turnover and improved anaerobic fitness with the restoration of normoxia.

**Abstract:**

On-growing juveniles of gilthead sea bream were acclimated for 45 days to mild-hypoxia (M-HYP, 40–60% O_2_ saturation), whereas normoxic fish (85–90% O_2_ saturation) constituted two different groups, depending on if they were fed to visual satiety (control fish) or pair-fed to M-HYP fish. Following the hypoxia conditioning period, all fish were maintained in normoxia and continued to be fed until visual satiation for 3 weeks. The time course of hypoxia-induced changes was assessed by changes in blood metabolic landmarks and muscle transcriptomics before and after exhaustive exercise in a swim tunnel respirometer. In M-HYP fish, our results highlighted a higher contribution of aerobic metabolism to whole energy supply, shifting towards a higher anaerobic fitness following normoxia restoration. Despite these changes in substrate preference, M-HYP fish shared a persistent improvement in swimming performance with a higher critical speed at exercise exhaustion. The machinery of muscle contraction and protein synthesis and breakdown was also largely altered by mild-hypoxia conditioning, contributing this metabolic re-adjustment to the positive regulation of locomotion and to the catch-up growth response during the normoxia recovery period. Altogether, these results reinforce the presence of large phenotypic plasticity in gilthead sea bream, and highlights mild-hypoxia as a promising prophylactic measure to prepare these fish for predictable stressful events.

## 1. Introduction

Reduced oxygen (O_2_) availability generates physiological and anatomical changes that increase ventilation rates, erythropoiesis, and tissue vascularization, with a decrease in muscle oxidative capacity and a switch in substrate preference towards more O_2_-efficient fuels [1,2,3]. These adaptive features occur across all the animal kingdom, contributing to epigenetic mechanisms to depress metabolic rates when individuals are facing predictable seasonal signals (hibernation, cold hardening, or diapause) or unpredictable episodic stresses, such as hypoxia, desiccation, or traumatic surgical situations [4,5,6]. Besides, epigenetics allows pre-programming of offspring to high-altitude hypoxic environments by imprinting genes at the embryonic or placental interface, resulting in transgenerational and/or intra-generational heritable changes that affect gene expression [7,8].

Hypoxia is also a common stressor in aquatic ecosystems [9,10,11] and “dead zones” expand rapidly in oceans as climate emergency causes unprecedented O_2_ losses [12,13]. This will have a strong negative impact on fisheries and aquaculture production [14], and future selective breeding will need to be directed towards more robust and resilient farmed fish to mitigate the effects of climate change [15,16]. The first sign of a mismatch between O_2_ supply and demand is the reduction of appetite, varying the O_2_ threshold level for maximal feed intake in Atlantic salmon (*Salmo salar*) and gilthead sea bream (*Sparus aurata*) between 40% and 75% saturation within the range of temperature tolerance [17,18]. This threshold level is decreased at high stocking densities [19,20,21], probably due to the unbalanced production and scavenging of reactive O_2_ species (ROS) [22]. However, acclimation to one stressor can also improve the capacity to cope with another critical co-occurring stressor, and warm acclimation improves the hypoxia tolerance in Atlantic killifish (*Fundulus heteroclitus*) [23]. Meanwhile, cold exposure also facilitates hypoxia adaptation, as the reduction of metabolic rates is likely accompanied by a reduction in mitochondrial O_2_ use [24]. These different metabolic strategies to cope with changing temperature and reduced O_2_ availability are also evidenced on a seasonal and developmental basis [25]. Thus, European sea bass (*Dicentrarchus labrax*) cope with moderate hypoxia at the expenses of a delayed larval maturation of digestive function [26]. Likewise, early acute hypoxia has transgenerational impairment effects on the reproductive performance of medaka (*Oryzias latipes*) [27]. By contrast, mild-hypoxia exposure during the embryonic development of zebrafish (*Danio rerio*) is protective against severe hypoxia insults later in life [28].

It is important to note that hypoxia acclimation affects endurance training in athletes and other animal models, including fish, usually via increased O_2_ uptake capacity and aerobic metabolic capacity [29,30,31]. In juveniles and fingerlings of farmed gilthead sea bream, successful adaption to severe and moderate hypoxia has been demonstrated to occur by the induction of hypo-metabolic states, increased O_2_-mitochondria affinity, and/or aerobic/anaerobic metabolic switches in substrate preference as metabolic fuels [20,32,33,34]. Thus, the goal of the present study is to underline new insights on the mild-hypoxia driving force for reprograming growth and swimming performance of on-growing juveniles of gilthead sea bream in order to prepare individuals to better respond to predictable stresses. For this purpose, the time course of metabolic responses after mild-hypoxia conditioning and normoxia recovery periods was assessed by changes in blood metabolic landmarks and muscle transcriptomics before and after exhaustive exercise in a swim tunnel respirometer.

## 2. Materials and Methods

### 2.1. Ethics Statement

All procedures were approved by the Ethics and Animal Welfare Committees of the Institute of Aquaculture Torre de la Sal (IATS) and CSIC. The study was conducted in the IATS’s registered aquaculture infrastructure facility (code ES120330001055), in accordance with the principles published in the European Animal Directive (2010/63/EU) and Spanish laws (Royal Decree RD53/2013) for the protection of animals used in scientific experiments.

### 2.2. Experimental Setup of Hypoxia Conditioning

**Figure 1:**
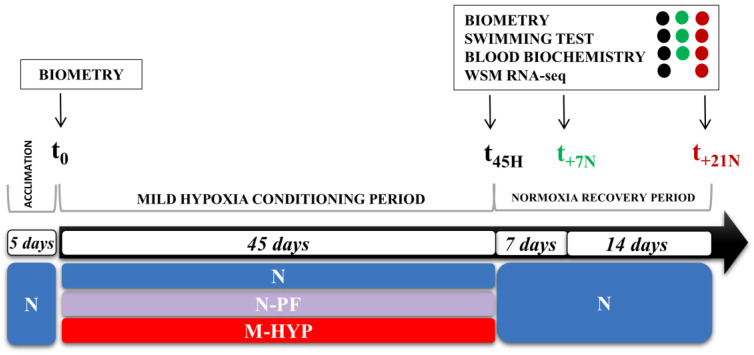
Experimental setup showing the timing and type of data recorded at each sampling point.

Gilthead sea bream juveniles of Atlantic origin (Ferme Marine du Douhet, Bordeaux, France) were reared from early life stages (3–5 g initial body weight) in the indoor experimental facilities of the Institute of Aquaculture Torre de la Sal (IATS, CSIC, Spain) under the natural photoperiod and temperature conditions at our latitude (40°5′ N; 0°10′ E). In June 2017, fish of 21–28 g body weight were randomly distributed in twelve 90 L tanks (n = 20), connected to two separated recirculating aquaculture systems (RAS) with regulation of the water temperature (24–26 °C) and O_2_ concentration (Supplementary Figure S1). As shown in Figure 1, fish were allowed to acclimate to experimental tanks for 5 days before any manipulation of O_2_ concentration, keeping the unionized ammonia below 0.02 mg/L. After this acclimation period, the O_2_ concentration of one RAS (six 90 L tanks) was ramped through 20 h to achieve a mild-hypoxia condition (M-HYP: 3–4 ppm, 40–60% O_2_ saturation), according to the values of limiting oxygen saturation (LOS, defined as O_2_ levels where the maximal metabolic rates start to decrease with further reduction in dissolved O_2_) reported for this fish species [17] at a given temperature. The remaining fish, coupled to a second RAS, were maintained under normoxic conditions (5.5–6 ppm, 85–90% O_2_ saturation). These fish constituted two different normoxic groups, depending on if they were fed to visual satiation (N) or pair-fed (N-PF) to the M-HYP group, fed to visual satiation, with a commercial diet (EFICO YM 853 3 mm, BioMar, Palencia, Spain) once daily (12:00 a.m., six days per week).

After 45 days of mild-hypoxia conditioning (t_45H_), 12 overnight-fasted fish per experimental condition were randomly selected and anesthetized (between 10:00 and 12:00 a.m.) with 100 mg/L 3-aminobenzoic acid ethyl ester (MS-222, Sigma, Saint Louis, MO, USA). Blood was taken from caudal vessels with heparinized syringes in less than 3 min for all the fish from the same tank. The haematocrit (Ht) and haemoglobin concentration (Hb) were determined in fresh samples. The remaining blood was centrifuged at 3000× *g* for 20 min at 4 °C, and plasma samples were frozen and stored at −20 °C until biochemical and hormonal analyses were performed. Prior to skeletal muscle collection, fish were killed by cervical section and representative portions of the dorsal tissue were excised and immediately snap-frozen in liquid nitrogen and stored at −80 °C until extraction of total RNA and tissue lactate quantification. At this stage, 6–7 additional fish per experimental condition were used for swim tests (see Section 2.3), and blood and skeletal muscle were rapidly taken from exhausted fish for biochemical and transcriptomic analyses. The remaining fish were kept under normoxia and continued to be fed until visual satiation for three additional weeks, which constituted the normoxia restoration period with additional sampling points at Week 1 (t_+7N_; 6 fish) and Week 3 (t_+21N_; 7 fish) for swim tests as well as biochemical and transcriptomic analyses. Data on body weight were retrieved for all fish at t_0_, t_45H_, t_+7N_, and t_+21N_ (see Figure 1).

### 2.3. Swim Tunnel Respirometer

Fish were exercised during hypoxia and normoxia restoration periods in an intermittent-closed swim tunnel respirometer of 10 L water volume (Loligo® Systems, Viborg, Denmark), as reported elsewhere [20,31]. To ensure a high water quality, the water bath was connected to a RAS with water temperature and O_2_ concentration set at 26 ± 0.5 °C and 60% saturation (4 ppm), respectively. For the testing procedures, slightly anesthetized fish were transferred into the swim tunnel, after obtaining their biometrical parameters, and recovered and acclimated at a swimming speed of 0.5–1.0 body lengths per second (BL/s). Acclimation was achieved when the O_2_ consumption rates (MO_2_) reached a constant low plateau, which typically happened after 30–45 min with an MO_2_ around 220–240 mgO_2_/kg/h [35]. After this acclimation period, the water velocity was increased in 0.5 BL/s steps, and fish were submitted to controlled speeds until exhaustion. Each swimming interval at a given velocity lasted 5 min, consisting of “flush–wait–measurement” cycles (60 s flush interval to exchange the respirometer water = “flush”; 30 s mixing phase in closed mode = “wait”; and a 210 s MO_2_ measuring period in closed mode = “measurement”). During the measurement interval, O_2_ saturation of the swim tunnel water was recorded every second. MO_2_ was automatically calculated by the AutoResp^TM^ software from linear decreases (r^2^ = 0.98–1.0) in chamber O_2_ saturation during the measurement period at each discrete and specific speed, using the appropriate constants for O_2_ solubility in seawater (salinity, temperature, and barometric pressure).

### 2.4. Blood Biochemistry

Haemoglobin (Hb) concentration was determined with a HemoCue B-Haemoglobin Analyser® (AB, Leo Diagnostic, Sweden). The haematocrit (Hc) was measured after centrifugation of blood in heparinized capillary tubes at 13,000× *g* for 10 min in a Sigma 1-14 centrifuge (Sigma). Blood lactate was measured in deproteinized samples (8% perchloric acid) by an enzymatic method based on the use of lactate oxidase and peroxidase (Ref. 1001330; SpinReact S.A., Girona, Spain). The same kit was used to determine muscular lactate concentrations after mincing and homogenization of samples by mechanic disruption in 7.5 volumes ice-cold 0.6 N perchloric acid, neutralized using 1 M KCO_3_, and centrifuged at 3000× *g* for 30 min at 4 °C. Plasma glucose was determined by the glucose oxidase method (ThermoFisher Scientific, Waltham, MA, USA) according to the manufacturer’s instructions. Plasma triglycerides (TAGs) were determined using lipase/glycerol kinase/glycerol-3-phosphate oxidase reagent. Plasma free fatty acids (FFA) were analysed using a commercial enzymatic method (NEFA-C, Wako Test, Neuss, Germany). Plasma cortisol levels were measured with a commercial Cortisol Enzyme Immunoassay Kit from Arbor Assays^TM^ (NCal^TM^ International Standard Kit, DetectX®, K003; Ann Arbor, MI, USA), following the manufacturer’s instructions. Plasma growth hormone (Gh) was determined by a homologous gilthead sea bream radioimmunoassay (RIA) [36]. Plasma insulin-like growth factor-1 (Igf-1) was extracted by acid-ethanol cryoprecipitation, and its concentration was determined by means of a generic fish Igf-1 RIA validated for Mediterranean perciform fish [37].

### 2.5. Illumina Sequencing and Sample Quality Assessment

Total RNA from tissue portions of white skeletal muscle was extracted using the MagMAX^TM^-96 for Microarrays total RNA isolation kit (Life Technologies, Carlsbad, CA, USA). The quality and integrity of the isolated RNA was checked on an Agilent Bioanalyzer 2100 total RNA Nano series II chip (Agilent, Santa Clara, CA, USA) with RIN (RNA Integrity Number) values varying between 8 and 10. Illumina RNA-seq libraries were prepared from 500 ng total RNA using the Illumina TruSeq™ Stranded mRNA LT Sample Prep Kit (Illumina Inc. San Diego, CA, USA) according to the manufacturer’s instructions. All RNA-seq libraries were sequenced on an Illumina HiSeq2500 sequencer as a 1 × 75 nucleotides single-end (SE) read format, according to the manufacturer’s protocol. Raw sequenced data were deposited in the Sequence Read Archive (SRA) of the National Center for Biotechnology Information (NCBI) under the Bioproject accession number PRJNA679473 (BioSample accession numbers: SAMN16834555-597). Approximately 882 million SE reads were obtained from the 50 samples sequenced, with an average of ~18 million reads per sample. Quality analysis was performed with FASTQC v0.11.7 (https://www.bioinformatics.babraham.ac.uk/projects/fastqc/ accessed on 27 April 2019), and libraries were filtered with Prinseq [38] for quality > 28 and < 5% of Ns in the sequence. Then, libraries were mapped and annotated using TopHat2 [39] and the gilthead sea bream draft genome as reference [40]. A representative transcriptome per sample was constructed using Cufflinks, with the data quality checked with CummeRbund [41].

### 2.6. Statistics

Changes in the growth performance and blood parameters through all the experiment were analysed by t-test or one-way ANOVA followed by Student–Newman–Keuls post-test. At t_45H_ and t_+21N_, the differentially expressed (DE) genes were retrieved with normalized fragment per kilobase per million (FPKM) values using Cuffdiff [41], with false discovery rates (FDR) adjustment with a cut-off of 0.05. To increase the number of DE genes without loss of statistical robustness, supervised partial least-squares discriminant analysis (PLS-DA) and hierarchical clustering of samples were sequentially applied using EZinfo v3.0 (Umetrics, Umea, Sweden) and the R package gplots, respectively. The genes included in this analysis were filtered by ANOVA *p*-values < 0.05. The final list of genes contributing to group separation was determined by the minimum Variable Importance in the Projection (VIP) values [42,43], driving the right clustering of all individuals in the heatmap analysis. To discard the possibility of over-fitting of the supervised discriminant model, a validation test consisting in 500 random permutations was performed using SIMCA-P+ v11.0 (Umetrics). The heatmap representation was constructed using the average linkage method and Euclidean distance. 

Genes above the VIP threshold were analysed for gene ontology (GO) with the R package ShinyGO v0.61 [44], after conversion of the gilthead sea bream annotated sequences to human equivalents. Significantly enriched GO categories were obtained after FDR correction using a cut-off of 0.05. The list of genes associated with enriched GO terms was introduced in the Search Tool for the Retrieval of Interacting Genes (STRING v.11) database [45]. Functional protein–protein association networks were considered statistically significant at FDR *p*-values < 0.05 and a high confidence score of 0.7. The tools used for the sequencing quality analysis, cleaning, mapping, transcriptome assembly, and differential gene expression are contained in the GPRO suite [46]. 

## 3. Results

### 3.1. Growth Performance during Mild-Hypoxia and Normoxia Restoration

Control fish (N group) grew during the hypoxia conditioning period (45 days), from 24 g to 79 g, at high specific growth rates (SGR = 2.59) for this species and class of size. Feed intake (g dry matter/fish) was reduced by 25% in fish exposed to mild-hypoxia, whereas the feed conversion ratio (FCR = dry feed intake/wet weigh gain) remained within optimum levels (0.98–0.95) in both N and M-HYP fish (Table 1). Normoxic pair-fed fish (N-PF) also grew efficiently (FCR = 0.96) at the same growth rate than M-HYP fish (SGR= 2.24–2.25%). During the first week of the subsequent normoxic and unrestricted feeding period (t_45H_–t_+7N_), the growth performance parameters remained similar in all groups, although during the last two weeks (t_+7N_–t_+21N_), growth rates of N-PF and M-HYP fish were higher (*p* < 0.001) than in control fish, as denoted by the SGR values (2.26 and 2.19 vs. 1.79, respectively), helping to compensate, at least in part, the initial growth impairment. Furthermore, the feed conversion ratio was improved to some extent (*p* = 0.103), with the achieved FCR varying between 1.21 in N fish to 1.13–1.14 in M-HYP/N-PF fish. 

### 3.2. Blood Patterns at the End of the Mild-Hypoxia Conditioning Period

Data on blood haematology and biochemistry in free-swimming fish at t_45H_ are shown in Table 2. In this fish group, the reduction of feed intake was associated to lowered (*p* = 0.011) Hb concentrations in N-PF fish, but control values were restored with the combined reduction of feed intake and O_2_ availability in M-HYP fish. Circulating levels of lactate were lowered in both N-PF and M-HYP fish, with the lowest levels in fish exposed to a low O_2_ concentration (*p* < 0.001). In contrast, feed intake and O_2_ availability showed an opposite effect on plasma levels of FFAs, achieving the highest concentrations in N-PF fish and the lowest in M-HYP fish (*p* = 0.029). No statistically significant differences were found in the other analysed parameters (Hc, glucose, TAGs, cortisol, Gh, Ifg-1), but the calculated Gh/Igf-1 ratio increased significantly (*p* < 0.05) from 0.13 in N fish to 0.25 in M-HYP fish.

### 3.3. Swim Tests: Critical Swimming and Blood Patterns after Exhaustive Exercise

**Figure 2:**
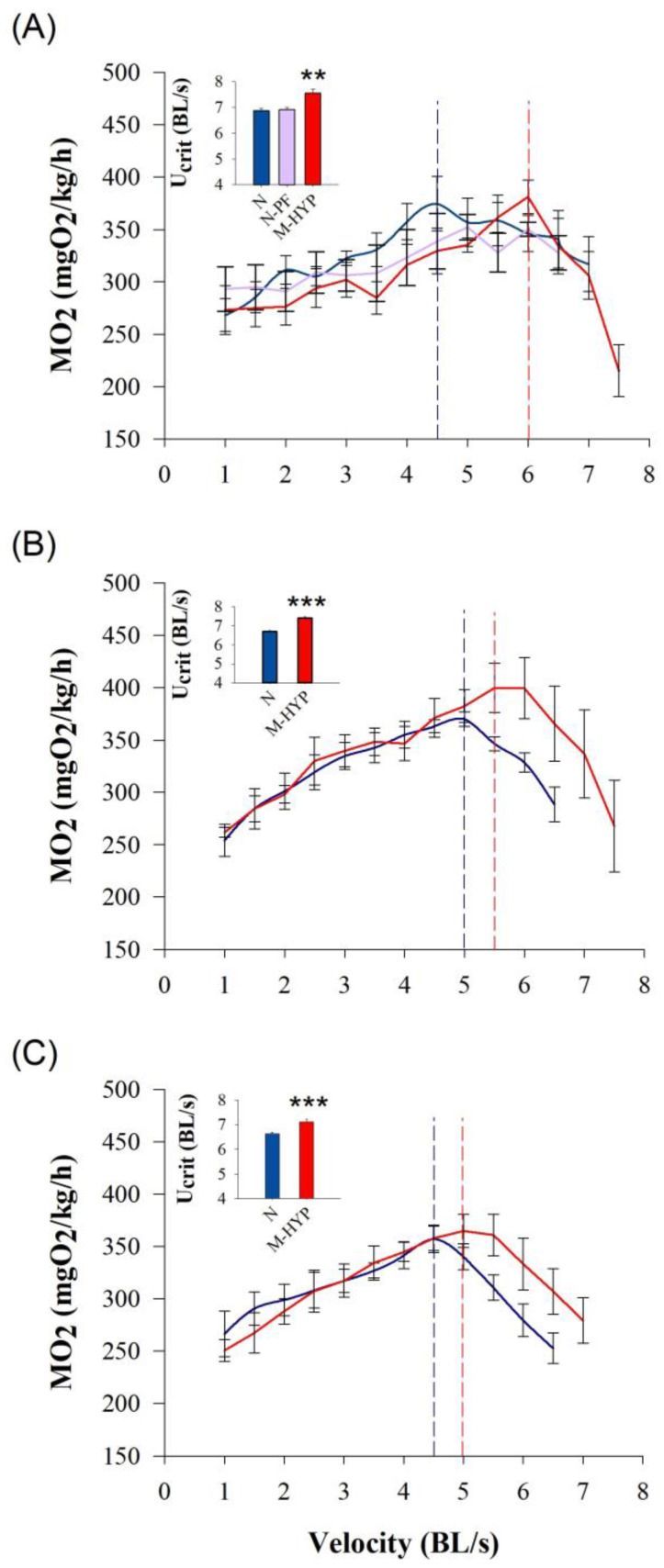
Swim tests conducted at the end of the mild-hypoxia conditioning period (t_45H_) (**A**), after a 1-week normoxia recovery period (t_+7N_) (**B**), and after a 3-week normoxia recovery period (t_+21N_) (**C**). Mild-hypoxia fish (M-HYP) are in red, pair-fed fish (N-PF) are in violet, and normoxic fish (N) are in blue. Values showing oxygen consumption (MO_2_), maximum metabolic Rate (MMR), and U_crit_ are the mean ± SEM of 4–6 fish. Asterisks indicate statistically significant differences among groups (one-way ANOVA, SNK test, ** *p* < 0.01; *** *p* < 0.001).

**Figure 3:**
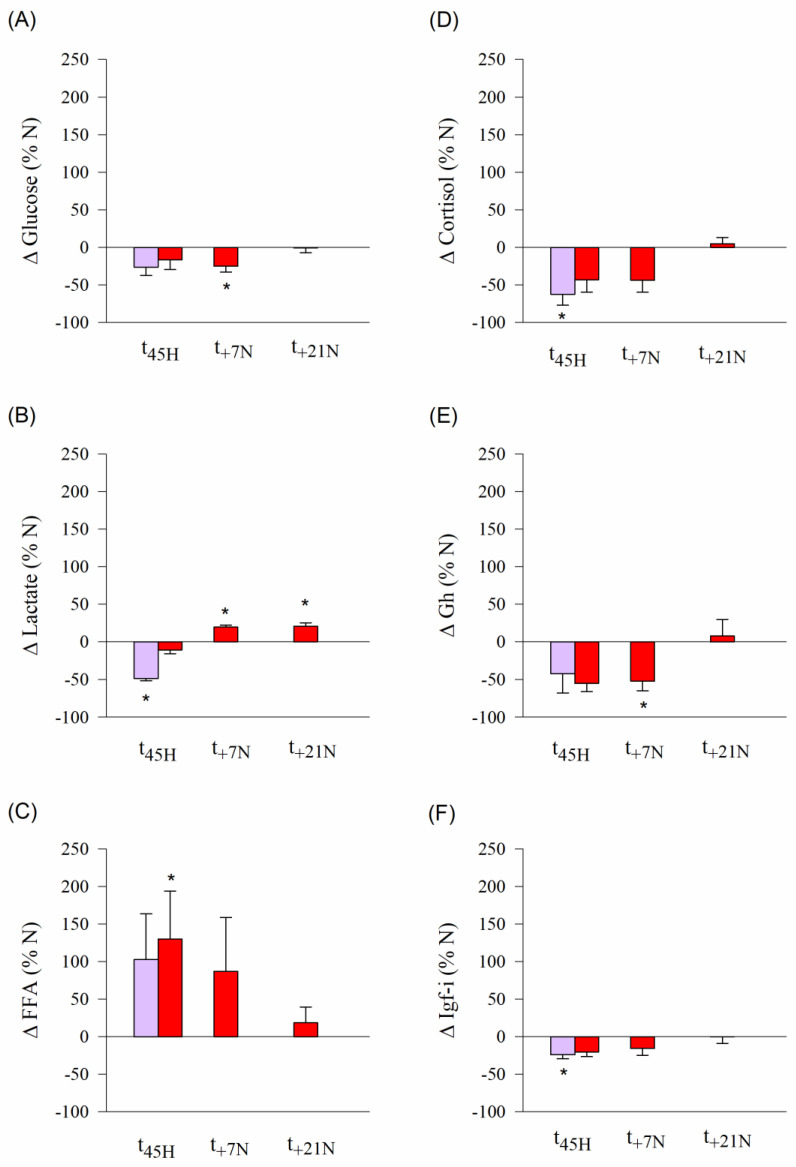
Changes in circulating levels of glucose (**A**), lactate (**B**), free fatty acids (**C**), cortisol (**D**), Gh (**E**), and Igf-I (**F**) in hypoxic (M-HYP, red) and pair-fed fish (N-PF, violet) following exhaustive exercise at the end of the mild-hypoxia conditioning period (t_45H_) and over the course of the normoxia recovery period (t_+7N_, t_+21N_). Values are the mean ± SEM of 4–6 fish. Asterisks indicate statistically significant differences with N fish at each experimental time (SNK test, * *p* < 0.05).

Results of the swim tests at different times over the experimental period (t_45H_, t_+7N_ and t_+21N_) are shown in Figure 2. Overall, MO_2_ increased linearly with the increase of water speed until a maximum metabolic rate (MMR) that varied non-significantly between 400 and 357 mgO_2_/kg/h. Then, fish of all experimental groups showed a sharp decrease in O_2_ consumption until being exhausted at their own critical speed (U_crit_). At t_45H_, the achieved U_crit_ was higher (*p* < 0.01) in M-HYP fish (7.6 BL/s) than in the other two experimental groups that shared undistinguishable critical swimming (6.8–6.9 BL/s) (Figure 2A). The subsequent swim tests at t_+7N_ and t_+21N_ were only conducted in control fish and M-HYP fish, which highlighted persistent higher U_crit_ values in M-HYP (*p* < 0.001) over the course of all the experimental period (Figure 2B,C). The effect of exhaustive exercise on circulating levels of metabolites and hormones in N-PF (t_45H_) and M-HYP fish (t_45H_, t_+7N_, t_+21N_) is shown as a percentage of change of N fish (Figure 3). Circulating levels of glucose, lactate, cortisol, Gh, and Igf-1 were lowered in N-PF and/or M-HYP in comparison to N fish at the end of the hypoxia conditioning period. However, this trend was reversed over time, especially in the case of lactate (*p* < 0.05). The opposite pattern was found for circulating FFAs, which shared raised levels in M-HYP fish at the beginning of the normoxia recovery period (*p* < 0.05), with a restoration of values of control fish at the last testing point (t_+21N_). Raw data on blood parameters are shown in Supplementary Table S1. 

### 3.4. Analysis of RNA-seq Libraries and DE Genes by Stringent FDR

**Figure 4:**
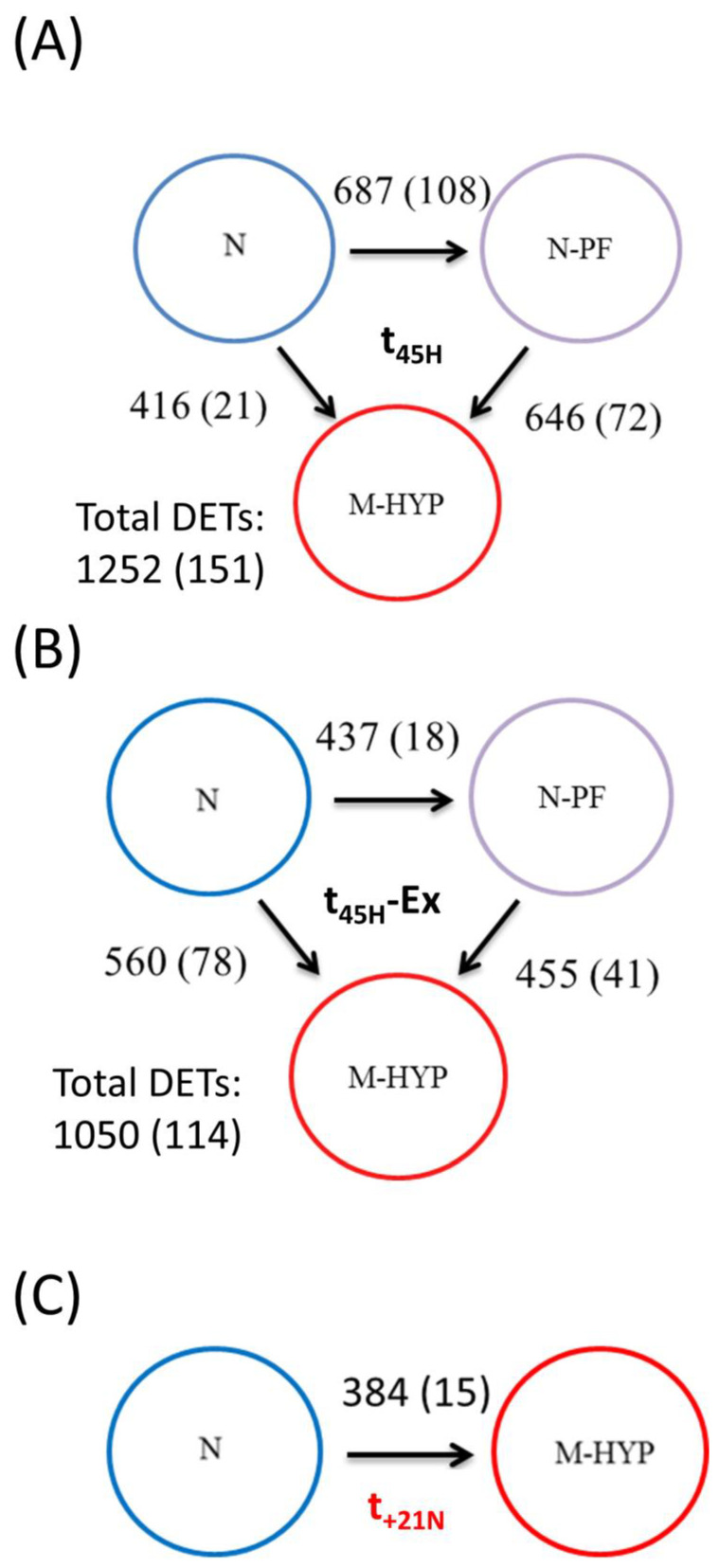
Differential expression analysis results at the end of the mild-hypoxia conditioning period (t_45H_) (**A**), after exhaustive exercise at t_45H_ (**B**), and after a 3-week normoxia recovery period (t_+21N_) (**C**). Numbers over or next to the arrows indicate differentially expressed genes (ANOVA, *p* < 0.05) between groups. Numbers between parentheses indicate differentially expressed genes (FDR-adjusted *p*-value < 0.05) between groups.

After trimming and quality filtering, around 3% of all skeletal muscle reads were discarded, with the remaining reads ranging between 91 million (6.83 Gb) and 121 million (9.08 Gb) in all experimental groups (see details in Supplementary Table S2). Up to 82% of these pre-processed reads were mapped against the reference genome, which retrieved 33,756 muscle transcripts. At t_45H_, 151 muscle transcripts (134 unique gene descriptions) were differentially expressed (FDR-adjusted *p*-value < 0.05) in free-swimming fish (Figure 4A). Among them, 108 genes were differentially regulated when comparisons are made between N-PF and N fish, decreasing these numbers to 72 and 21 transcripts when comparing M-HYP against N-PF fish, and M-HYP against N fish, respectively. After exercise exhaustion at t_45H_, the number of DE transcripts was 114 (101 unique gene descriptions) for an FDR-adjusted *p*-value < 0.05 (Figure 4B). Among them, 18 transcripts were differentially regulated when comparing N-PF and N fish, 41 when comparing M-HYP and N-PF groups, and 78 when comparing M-HYP and N fish. The magnitude of change was apparently decreased over time with only 15 DE transcripts (FDR-adjusted *p*-value < 0.05) when comparisons are made between M-HYP and N fish at t_+21N_ (Figure 4C).

### 3.5. Discriminant Classifiers and Enriched GO Terms

**Figure 5:**
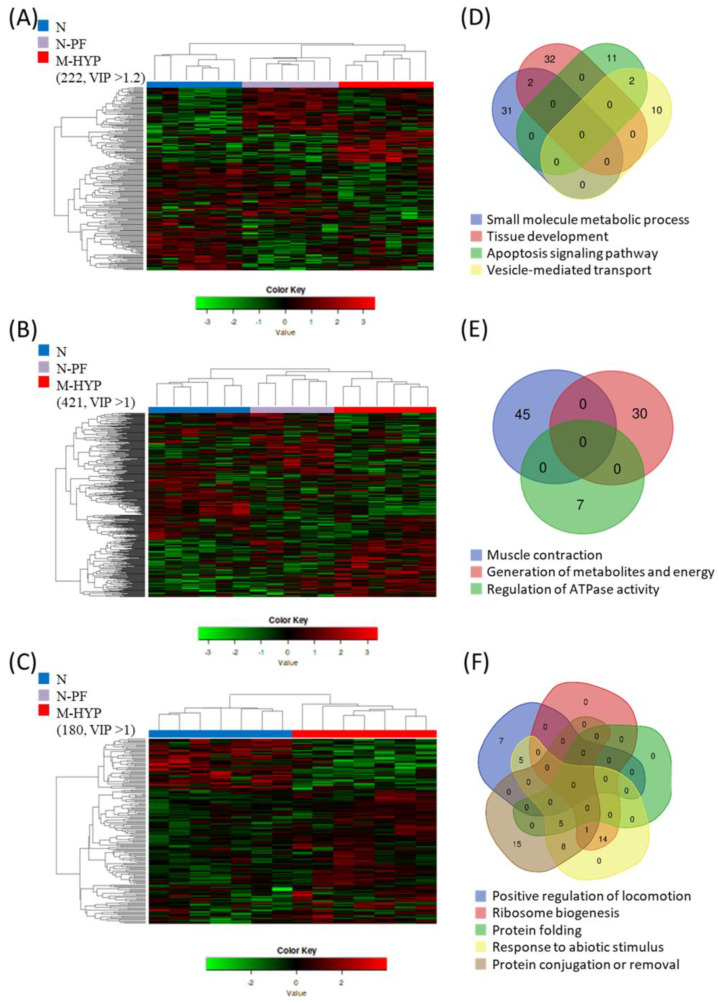
Clustering and gene enrichment analysis. Heatmap showing the abundance distribution (z-score) of the DE genes identified to be driving the separation between groups at the end of the mild-hypoxia conditioning period (t_45H_) before (**A**) and after exhaustion exercise (**B**), and at the end of the normoxia recovery period (t_+21N_) after exhaustion exercise (**C**). Venn diagrams show the level of overlapping of enriched GO-BP categories at the end of the mild-hypoxia conditioning period before (**D**) and after exhaustion exercise (**E**), and at the end of the normoxia recovery period after exhaustion exercise (**F**).

For a given sampling time, supervised PLS-DA models of the skeletal muscle transcriptome clearly separated along the X-axis the N fish from M-HYP fish (Supplementary Figure S2) in the analysis of free-swimming fish at t_45H_. Otherwise, the two first components explained more than 85% and 95% of total variance in forced exercise fish after conditioning (t_45H_) (Supplementary Figure S2A,B) and recovery (t_+21N_) (Supplementary Figure S2C,D,E,F), respectively. This classifier performance was validated by 500-model permutation tests (Supplementary Figure S3), which was reinforced by the right hierarchical clustering of samples when applying different cut-offs for the VIP values. At t_45H_, such an approach yielded two main clusters corresponding to N fish and N-PF/M-HYP fish. However, the number of DE transcripts among groups was increased from 222 (219 unique gene descriptions) to 421 (400 unique gene descriptions) by exhaustive exercise, decreasing in parallel the VIP cut-off value from 1.2 to 1 (Figure 5A,B). The VIP cut-off for right clustering remained low at t_+21N_, but the number of DE transcripts between N and M-HYP fish decreased until 180 (179 unique gene descriptions) (Figure 5C). 

After gene clustering, a functional enrichment analysis was performed at each sampling time. The enriched categories in Biological Processes (BP), together with their allocated genes annotation and expression values in each comparison, are shown in Supplementary Table S3. For mild-hypoxia in free-swimming fish (Figure 5D), the enriched processes were (1) small molecule metabolic process, mainly lipid metabolism (GO:0044281; 33 allocated genes); (2) tissue development (GO:0009888; 34 allocated genes); (3) apoptosis signalling pathway (GO:0097190; 13 allocated genes); and (4) vesicle-mediated transport, mainly endocytosis (GO:0016192; 12 allocated genes). After exhaustive exercise at t_45H_ (Figure 5E), the enriched processes were (1) muscle contraction (GO:0006936; 42 allocated genes); (2) generation of precursors of metabolites and energy (GO:0006091; 30 allocated genes); and (3) regulation of ATPase activity (GO:0043462; 7 allocated genes). After normoxia recovery and exhaustive exercise at t_+21N_ (Figure 5F), the enriched processes were (1) positive regulation of locomotion (GO:0040017; 12 allocated genes); (2) ribosome biogenesis (GO:0042254; 15 allocated genes); (3) protein folding (GO:0006457; 5 allocated genes); (4) response to abiotic stimulus (GO:0009628; 33 allocated genes); and (5) protein conjugation or removal (GO:0070647; 29 allocated genes).

### 3.6. Linking Enriched Processes with Gene Expression Patterns

**Figure 6:**
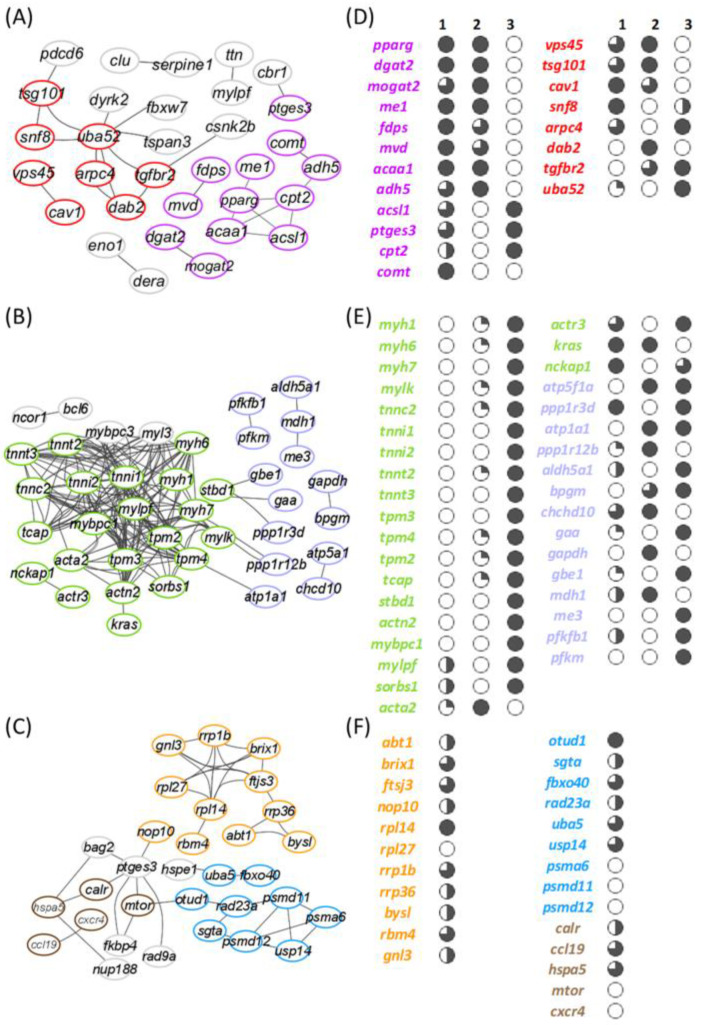
Protein–protein interaction plots and expression patterns of enriched processes at the end of the mild-hypoxia conditioning period (t_45H_) before (**A**,**D**) and after exhaustive exercise (**B**,**E**), and at the end of the normoxia recovery period (t_+21N_) after exhaustion exercise (**C**,**F**). Edges between nodes shows significant relations (FDR < 0.05; STRING confidence score > 0.7). Colours represent genes related to the following enriched biological processes: small molecule metabolic process (purple), vesicle-mediated transport (red), muscle contraction (green), generation of precursors of metabolites and energy (violet), ribosome biogenesis (orange), small protein conjugation or removal (blue), and positive regulation of locomotion (brown). Numbers above columns indicate the comparison in the RNA-seq analysis (1: M-HYP vs. N; 2: M-HYP vs. N-PF; 3: N-PF vs. N). Sliced symbols in (**D**) and (**E**) represent the comparison between the gene expression log_2_FC values in the respective comparison for each gene. White circles represent the lowest log_2_FC values, whereas the black circles represent the highest. Genes in (**C**), forming a category in the interaction plot, were ordered by their log_2_FC in comparison 1, and sliced symbols in (**F**) were then applied for all the genes at the same time.

According to the protein–protein network analysis, a total of 31 interactions corresponding to 32 genes allocated to enriched processes were disclosed in free-swimming fish after the mild-hypoxia conditioning period (Figure 6A). One major link comprised molecules linked to endocytosis (KEGG: ko04144; 7 genes) and apoptotic signalling in response to DNA damage processes (GO:0008630; 6 genes), with ubiquitin-60S ribosomal protein L40 (*uba52*) connecting up to 8 genes involved in both processes. However, there is not a clear gene expression pattern associated with reduced O_2_ availability or restricted feed intake (Figure 6D). Conversely, most lipid-related genes of the interaction plot disclosed a clear upregulation in M-HYP fish. The peroxisome proliferator-activated receptor gamma (*pparγ*) worked as a pivotal molecule connecting up to 4 genes, all of them framed in the PPAR signalling pathway (KEGG: ko03320). This pivotal gene was significantly upregulated in the comparisons of M-HYP vs. N and M-HYP vs. N-PF. A similar expression pattern was found for diacylglycerol O-acyltransferase 2 (*dgat2*), 2-acylglycerol O-acyltransferase 2-A-like (*mogat2*), NADP-dependent malic enzyme (*me1*), farnesyl pyrophosphate synthase (*fdps*), diphosphomevalonate decarboxylase (*mvd*), 3-ketoacyl-CoA thiolase B, peroxisomal (*acaa1*), and alcohol dehydrogenase 5 (*adh5*). The long-chain-fatty-acid--CoA ligase 1 (*acsl1*) and prostaglandin E synthase 3 (*ptges3*) were equally upregulated in both M-HYP and N-PF fish in comparison to N fish. By contrast, in comparison to the controls, the expression of carnitine O-palmitoyltransferase 2 (*cpt2*) was activated in N-PF fish but not in M-HYP fish. 

After the swim test following mild-hypoxia conditioning, the number of connections increased up to 146 with 40 genes in the enriched GO terms (Figure 6B), which indicates an increased cohesion pattern between DE genes following stringent exercise. Besides, such physiological response rendered the interaction of 22 genes related to muscle contraction and sliding. In this sampling point, a strong overall downregulation of genes involved in muscular machinery was found in the comparisons M-HYP vs. N and M-HYP vs. N-PF, with no differences in the comparison N-PF vs. N (Figure 6E). Among them, myosin-1, -6 and -7 (*myh1*, *myh6*, *myh7*), myosin light chain kinase (*mylk*), troponin C (*tnnc2*), troponin I, slow and fast skeletal muscle (*tnni1*, *tnni2*), troponin T, cardiac and skeletal muscle isoform (*tnnt2*, *tnnt3*), and tropomyosin alpha (*tpm3*, *tpm4*) and beta (*tpm2*) chains were disclosed under this type of response. 

Following the normoxia recovery period (t_+21N_), targeted genes of the enriched BP were also changing, the number of interactions decreasing to 43 and corresponding to 31 genes (Figure 6C). One major link in this group involved a total of 11 genes related at the same time with ribonucleoprotein complex biogenesis and response to abiotic stimulus (Figure 6F). The generalized response of these genes was their upregulation in M-HYP fish in comparison to N fish. Among others, the activator of basal transcription 1 (*abt1*), the ribosome biogenesis protein BRX1 homolog (*brix1*), and the ribosomal RNA processing proteins 1B and 36 (*rrp1b*, *rrp36*) were found. A second link grouped 9 genes related with the protein modification by small protein conjugation or removal. Here, genes related with deubiquitination processes, as the OTU domain-containing protein 1 (*otud1*), the UV excision repair protein RAD23 homolog A (*rad23a*), the ubiquitin-like modifier-activating enzyme 5 (*uba5*), and the ubiquitin carboxyl-terminal hydrolase 14 (*usp14*), were upregulated in M-HYP fish. Otherwise, genes involved in the proteasome degradation such as the proteasome subunit alpha type-6 (*psma6*), the 26S proteasome non-ATPase regulatory subunit 11 (*psmd11*), and the 26S proteasome non-ATPase regulatory subunit 12 (*psmd12*) were downregulated in M-HYP fish. A third important link was found between 5 genes related to the regulation of locomotion and the response to abiotic stimulus with the upregulation of calreticulin (*calr*), C-C motif chemokine 19 (*ccl19*), and heat shock 70kDa Protein 5 (*hspa5*/*grp78*), and the downregulation of serine/threonine-protein kinase mTOR (*mtor*) and C-X-C chemokine receptor type 4 (*cxcr4*) in M-HYP fish.

## 4. Discussion

Episodes of high temperature and hypoxia are increasing in extent and severity in coastal marine ecosystems, and these stressors have the capacity to reinforce each other because increasing temperature decreases O_2_ solubility [47]. Hypoxia is, thereby, a major aquaculture stressor around the world [48]. The first sign of O_2_ scarcity is the reduction of appetite, and subsequent growth impairments reflect the different temperature and O_2_ tolerance ranges of living organisms [18,49,50,51], as well as their plasticity for prioritizing feed efficiency at the expenses of maximum growth “oxystatic theory” [52,53]. Thus, both in this and a previous study [20], we found that mild-hypoxia acclimation in summer (40–60% saturation) deaccelerated growth of fast-growing juveniles of gilthead sea bream, whereas FCR was not impaired or even improved during mild-hypoxia and normoxia recovery periods, respectively. This is because the best feed conversion and hormonal signatures for fast and efficient growth generally occur before the achievement of maximum growth at the greater ration size [54,55]. This also applies at the cellular level, where the maximum ATP yield per molecule of O_2_ (P/O ratio) is increased during food shortage [56,57] or hypometabolic hypoxia [33]. Such adaptive feature was supported herein by lowered plasma levels of lactate, which would reflect a reduced basal metabolism rather than a switch of aerobic to anaerobic metabolism during mild-hypoxia exposure. This was also previously stated [20], but herein the pair-fed experimental design allowed us to disclose that low plasma lactate levels arise from a reduced feed intake that becomes slightly although non-significantly lowered by limited O_2_ availability. Conversely, circulating levels of haemoglobin and FFAs evolved differentially when fish faced changes in feed intake and O_2_ availability, resulting in reduced erythropoiesis and enhanced lipid mobilization, which can be interpreted as a discriminant feature of hypo-metabolic states triggered by feed restriction during normoxia. Otherwise, circulating levels of cortisol and growth-promoting factors did not differ significantly among groups, though the highest Gh/Igf-1 ratio of M-HYP fish during hypoxia exposure underlines a limited growth potentiality under reduced O_2_ and metabolic fuels availability [58].

The changing metabolic phenotype is also indicative of a number of whole-organism traits of ecological and economic importance, such as dominance, aggression, and swimming performance [59]. A high percentage of this genetic variance is expected to be unexplained [60], but recent research in gilthead sea bream associated reduced locomotor activity with more continuous growth [61], less size heterogeneity [34,61], and enhanced phenotypic plasticity of gut microbiota [62]. Likewise, large domesticated strains of Atlantic salmon and rainbow trout (*Oncorhynchus mykiss*) become athletically less robust that wild fish [63,64,65,66]. However, the opposite is also true and different exercise protocols are able to increase the growth performance of a wide range of farmed fish, including gilthead sea bream [67,68,69]. Moreover, critical swimming is a highly predictive marker of fillet yield in both gilthead sea bream and Atlantic salmon [70]. The mechanisms by which hypoxia acclimation or hypoxia priming during early life affect fillet yield remain elusive, though the present study highlighted that M-HYP fish showed a higher U_crit_ than the two other experimental groups. This observation underscored the improved availability of this group of fish to resist fatigue during training endurance, as it has been reported for hypoxia acclimation in athletes and different animal models [29,30]. Interestingly, exercised M-HYP fish would benefit of this metabolic advantage through all the normoxia recovery period (21 days), though the time course of changes in blood landmarks evidenced a dynamic metabolic trade-off with an increased availability of aerobic metabolic fuels (increase of circulating levels of FFA for aerobic ATP production) after hypoxia conditioning, which shifted towards a higher anaerobic fitness (increased lactate production from glucose) at the end of the normoxia recovery period. 

At the transcriptional level, up to 222 genes have a discriminant role for classification of all individuals in their respective group (N, N-PF, or M-HYP) at the end of the hypoxia conditioning period. During hypoxia exposure, the number of discriminant genes was also increased up to 421 by exhaustive exercise, which is in line with previous studies in rainbow trout where sustained swimming increased the transcriptional activity of skeletal muscle [71]. Certainly, in our experimental model, the number of interactions and interacting genes in the protein interaction plots increased largely in exercised fish. In any case, cluster analysis grouped together N-PF and M-HYP fish before and after exhaustive exercise, which indicates that most hypoxia-mediated changes in mRNA transcripts were mediated, at least in part, by a reduced feed intake. This assumption was supported by a high representation of discriminant genes (68–70%) that were up- or downregulated in parallel in both N-PF and M-HYP fish (Supplemental Table S3).

At a closer look, the protein interaction plots disclose lipid metabolism (small molecule metabolic process) as a main differentially regulated process in our hypoxia model (Figure 6A,D). This is not surprising given that many aspects of carbohydrates, but also of lipid metabolism, are modified in humans and transgenic animals by HIF (hypoxia inducible factor) during physiological or pathological hypoxia, contributing significantly to the pathogenesis and/or progression of cancer and metabolic disorders [72]. The ultimate mechanisms remain controversial, though experimental evidence supports that TAG synthesis and the extracellular uptake of FAs are promoted under hypoxia by the transcription factor PPARγ in a HIF-dependent manner [73,74]. Lipid accumulation under hypoxia is further supported by the HIF inhibition of FA oxidation via the downregulation of the transcriptional coactivator PGC-1α (proliferator-activated receptor-γ coactivator-1α) [75,76]. Thus, a number of studies have shown that obesity is increased by inhibition of HIF and decreased by HIF activation [77], but other authors claimed that HIF activation induces obesity [78,79]. Indeed, the activation of both lipid catabolism and anabolism was co-occurring in the present study, the hypoxic induction of *pparg* being associated with the upregulation of *acaa1* (peroxisomal β-oxidation enzyme) and *acsl1*, which catalyses the conversion of long-chain fatty acids to their active form acyl-CoAs for both oxidation and biosynthesis processes. This hypoxic activation of lipid anabolism was also supported by the upregulation of key-rate limiting enzymes of FA synthesis (*me1*) and esterification into diacylglycerols (*mogat2*) and TAG (*dgat2*). A similar enhancement of lipid metabolism at brain, liver and muscle has been recently reported in Ya fish *Schizothorax prenanti* as an adaptation to acute hypoxia stress, together with an upregulation of antioxidant genes [80]. This overall activation of lipid metabolism reflects an adaptive trade-off that might serve to facilitate the aerobic energy metabolism during prolonged reduced O_2_ availability, limiting at the same time the risk of lipotoxicity (excess of intracellular FAs) through the induction of TAG accumulation [81,82]. Thus, the muscle expression of *pparg* and other lipogenic/anabolic enzymes (*me1*, *mogat2*, *dgat2*) was repressed in N-PF, whereas the trend for catabolic enzymes of peroxisomal (*acsl1*) and mitochondrial (*cpt2*) β-oxidation was the upregulation in N-PF fish and to a lower extent in M-HYP fish. In other words, the activation of lipid cell storage would be primarily mediated by O_2_ availability, whereas the overall stimulation of lipid catabolic enzymes would be triggered by a deficit in metabolic fuels. In this line, hypoxia induced a pronounced mobilization of stored TAGs in the euryoxic goby *Gillichthys mirabilis* [83]. Otherwise, the activation of the transcription factor SREPB (sterol regulatory element binding protein) signalling pathway was required in the poor survival glioblastoma multiforme to preserve lipid biosynthesis and cell viability under lipid- and O_2_-deprived conditions [84], which was herein in agreement with the upregulated expression of *fdps* and *mvd* in both N-PF and M-HYP fish.

The hypoxic induction of *pparg* persisted after exhaustive exercise in M-HYP fish at the t_45H_ sampling point. However, protein interaction plots rendered the over-representation of the two other main processes (muscle contraction and generation of metabolites and energy), with an overall downregulation in comparison to normoxic control fish that was extensive to a lower extent in N-PF fish (Figure 6B,E). This metabolic feature highlighted the more efficient energy metabolism of M-HYP fish, probably with a higher contribution of aerobic metabolism to whole energy supply, as part of the mechanisms of a wide range of animal taxa to cope with high-altitude hypoxia [85,86,87,88,89,90,91]. The precise mechanisms remain unsolved in our experimental model, though the downregulated expression of muscle lactate dehydrogenase should contribute to regulate muscle contractions. Indeed, several studies stated that lactate decreases the blood flow to the working muscle, in a process that induces fatigue [92,93]. Marathoners deal with this fatigue with a higher proportion of oxidative fibres, which contract slowly and use aerobic respiration to produce ATP [94,95]. This type of adaptive response might also contribute to drive the muscle transcriptome of M-HYP fish, resulting in a persistent increase in critical swimming speed, due to their improved endurance training during the hypoxia conditioning period. Reinforcing this assumption, mRNA transcripts of myosin, troponin, and tropomyosin were strongly downregulated in M-HYP fish, but remained unaltered in N-PF fish, which suggests that this feature is a good example of a muscle transcriptional response mediated by changes in O_2_ availability rather than feed intake.

From our results, it was also conclusive that normoxic M-HYP fish (t_+21N_) shared a catch-up growth response, in concurrence with a persistent improvement in the swimming performance and changing muscle transcriptome after exercise exhaustion. These hypoxia-driven effects included a clear exercise activation of the anaerobic glycolysis pathway in M-HYP fish in comparison to normoxic control fish (Supplemental Table S3). Certainly, the regulation of glycolysis has been largely studied in fish [96,97,98], and wide-transcriptomic studies highlighted a fast transition from aerobic oxidation to anaerobic glycolysis when individuals faced restrictive O_2_ concentrations [99]. However, anaerobic metabolism is less efficient than aerobic ATP production, and the enhanced exercise activation of glycolysis by hypoxia conditioning was apparently delayed over the course of the normoxia recovery period. Intriguingly, this glycolysis activation was supported by increased levels of blood lactate (Figure 3B) and the upregulated expression of muscle lactate dehydrogenase. However, the protein interaction plot showed the main enrichment of ribosome biogenesis and protein conjugation GO terms with the upregulation of markers of protein synthesis (*abt1*, *brix1*, *rrp1b*, *rrp36*) and deubiquitination (*otud1*, *rad23a*, *usp14*) pathways, in combination with the strong downregulation of several proteasome subunits (*psma6*, *psmd11*, *psmd12*) (Figure 6C,F). This finding highlighted a reduced muscle protein turnover after exhaustive exercise in normoxic M-HYP, which would also contribute to support the catch-up growth of N-PF and M-HYP fish during the normoxia recovery period. Indeed, a large body of evidence has revealed that faster growing and/or more efficient fish have lower rates of protein turnover (equivalent to protein breakdown in growing individuals) [100,101]. Otherwise, a number of overlapping genes for positive regulation of locomotion and response to stimulus were also induced or repressed by exhaustive exercise in M-HYP fish. Among them, noteworthy is the upregulated expression of *hspa5/grp78* and *calr,* highly conserved chaperone proteins of endoplasmic reticulum (ER) that reduce ER stress and apoptosis through the enhancement of the cellular folding capacity [102,103]. Certainly, micro-RNAs (miRs) have emerged as key gene regulators in many diseases, as reduced miR30a increased the HSPA5 level and attenuated ischemic brain infarction in focal ischemia stroked mice [104]. Likewise, both *hspa5* and *calr* belong to the antioxidant defence system of the epithelial layers [105,106], and their upregulated expression in M-HYP fish would contribute to prevent the disruption of the intracellular redox state and the cellular folding capacity of skeletal muscle under metabolic states, resulting in high O_2_ consumption rates and enhanced ROS production.

## 5. Conclusions

**Figure 7:**
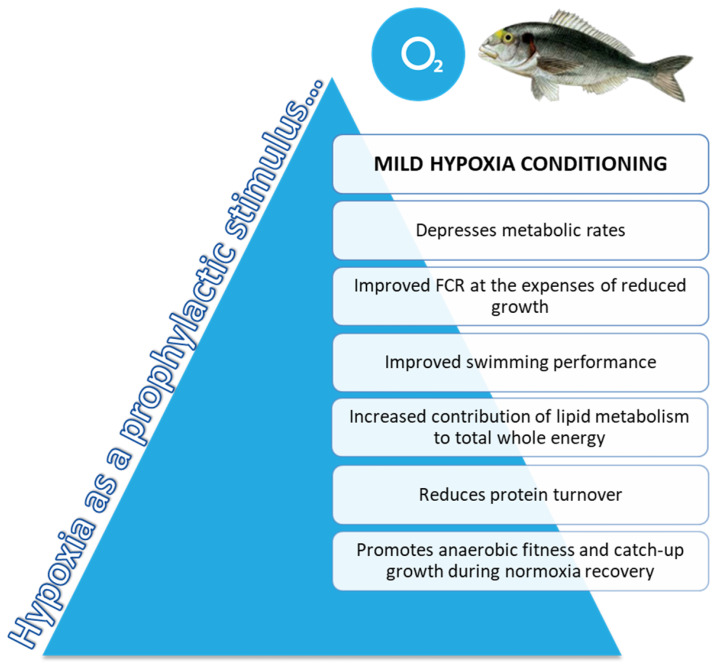
Schematic representation of the proposed model for integrative responses of gilthead sea bream exposed to mild-hypoxia stress conditioning.

Mild-hypoxia acclimation acts as a driving force with effects on growth and swimming performance through changes in metabolic and muscle transcriptomic landmarks. The first consequence of mild-hypoxia exposure was to prioritize feed efficiency at the expenses of maximum growth. Such adaptive feature would take advantage of the benefits of a reduced feed intake and hypometabolic state, resulting in a higher contribution of aerobic metabolism to whole energy supply that shifted towards a higher anaerobic fitness following normoxia restoration. Despite the changes in substrate preference for metabolic fuels, mild-hypoxia acclimation led to higher U_crit_ at exhaustive exercise before and after normoxia restoration, which would reflect the metabolic imprint of a lower O_2_ availability rather than a reduced feed intake in our hypoxia pair-fed model, at least in the short/medium-term (21 days post-recovery). At the transcriptomic level, our results depicted the overall activation of lipid metabolism to facilitate the aerobic energy metabolism during prolonged reduced O_2_ availability, with the limitation at the same time of the risk of lipotoxicity through the enhanced TAG accumulation of the excess of intracellular FAs. The machinery of muscle contraction and protein synthesis and breakdown was also largely altered by mild-hypoxia conditioning, and the achieved responses contributed to mitigate fatigue response under exhaustive exercise, and to preserve a catch-up growth during the normoxia recovery period. Altogether, these results reinforce the high phenotypic plasticity of gilthead sea bream, which is supported at the genomic level by a high rate of recent local gene duplications [40] that might favour the acquisition of novel gene functions, and a rapid and efficient adaption of individuals to a changing and challenging environment. In a practical sense, as summarized in Figure 7, mild-hypoxia pre-programming emerges as a promising prophylactic measure to depress metabolic rates and so prepare individuals to respond to predictable stressful events, preserving and even improving FCR and swimming performance.

## Figures and Tables

**Table 1:** Effects of mild-hypoxia conditioning on the growth performance of gilthead sea bream juveniles in a 45-day trial followed by a normoxia recovery period of 21 days. Values are the mean ± SEM of triplicate tanks. The *p*-values are the result of one-way ANOVA. Different superscript letters indicate significant differences between the experimental groups (SNK test, *p* < 0.05).

	N	N-PF	M-HYP	*p*-Value
*Mild-hypoxia conditioning* (t_0_–t_45H_)				
Initial body weight (g)	24.58 ± 0.11	24.1 ± 0.10	24.19 ± 0.03	0.112
Final body weight (g)	78.69 ± 0.79 ^b^	66.13 ± 1.41 ^a^	66.06 ± 0.97 ^a^	<0.001
Feed intake (g DM/fish)	53.36 ± 0.15 ^b^	40.77 ± 0.22 ^a^	40.08 ± 0.84 ^a^	<0.001
Weight gain (%) ^1^	220.30 ± 2.03 ^b^	174.51 ± 4.50 ^a^	173.22 ± 3.86 ^a^	<0.001
SGR (%) ^2^	2.59 ± 0.01 ^b^	2.24 ± 0.04 ^a^	2.23 ± 0.03 ^a^	<0.001
FCR (%) ^3^	0.98 ± 0.009	0.96 ± 0.02	0.95 ± 0.008	0.285
*Normoxia recovery period* (t_+7N_–t_+21N_)				
Initial body weight (g)	98.76 ± 1.20 ^b^	83.50 ± 0.50 ^a^	82.00 ± 1.14 ^a^	<0.001
Final body weight (g)	126.5 ± 1.30 ^b^	114.7 ± 0.33 ^a^	111.3 ± 1.81 ^a^	0.001
Feed intake (g DM/fish)	37.62 ± 1.28	36.57 ± 1.50	35.66 ± 0.50	0.329
Weight gain (%) ^1^	28.54 ± 0.46 ^a^	37.22 ± 1.33 ^b^	35.50 ± 0.85 ^b^	0.001
SGR (%) ^2^	1.79 ± 0.03 ^a^	2.26 ± 0.07 ^b^	2.19 ± 0.04 ^b^	<0.001
FCR (%) ^3^	1.21 ± 0.03	1.12 ± 0.02	1.14 ± 0.02	0.103

^1^ Weight gain (%) = (100 × body weigh increase)/initial body weight; ^2^ Specific growth rate = 100 × (ln final body weight − ln initial body weight)/days; ^3^ Feed conversion ratio = dry feed intake/wet weight gain.

**Table 2:** Effects of mild-hypoxia conditioning on blood haematology and blood biochemistry of gilthead sea bream juveniles. Values are the mean ± SEM of 6–10 fish (2–3 fish per replicate tank). The *p*-values are the result of one-way ANOVA. Different superscript letters indicate significant differences between the experimental groups (SNK test, *p* < 0.05).

	N	N-PF	M-HYP	*p*-Value
Haemoglobin (g/dL)	8.36 ± 0.38 ^b^	6.43 ± 0.64 ^a^	7.88 ± 0.22 ^b^	0.011
Haematocrit (%)	34.7 ± 1.24	33.7 ± 0.99	31.0 ± 1.41	0.175
Lactate (mg/dL)	14.1 ± 0.15 ^b^	6.32 ± 0.57 ^a^	4.18 ± 0.77 ^a^	<0.001
Glucose (mg/dL)	57.1 ± 5.98	55.7 ± 2.29	56.8 ± 2.35	0.493
Triglycerides (mg/dL)	2.80 ± 0.28	4.02 ± 0.34	3.02 ± 0.46	0.128
Free fatty acids (nmol/µL)	0.426 ± 0.052 ^ab^	0.595 ± 0.045 ^b^	0.388 ± 0.045 ^a^	0.029
Cortisol (ng/mL)	24.1 ± 5.43	29.3 ± 10.56	14.3 ± 4.71	0.270
Growth hormone (ng/mL)	9.19 ± 3.94	12.4 ± 5.30	13.9 ± 4.87	0.752
Insulin-like growth factor-1 (ng/mL)	69.3 ± 5.74	60.5 ± 3.42	55.5 ± 3.94	0.285
Gh/Igf-1	0.13 ± 0.058 ^a^	0.20 ± 0.081 ^ab^	0.25 ± 0.041 ^b^	0.032

## Data Availability

The datasets presented in this study can be found in online repositories. The names of the repository/repositories and accession number(s) can be found below: NCBI (accession: SAMN16834555-597, PRJNA679473).

## Supplementary Materials

The following are available online at https://www.mdpi.com/article/10.3390/biology10050416/s1. Figure S1: Closed-system scheme for the experimental set-up. Figure S2: Graphical representation of the goodness-of-fit and scores of the PLS-DA model in each sampling time along the experiment. (A), (C) and (E) Graphical representation of the goodness-of-fit of the PLS-DA model of t_45H_, t_45H_ after exercise and t_+21N_ after exercise, respectively. (B), (D), (F) Two-dimensional PLS-DA score plot representing the distribution of the samples between the first two components in the model of t_45H_, t_45H_ after exercise and t_+21N_ after exercise, respectively. Figure S3: Validation plots of the PLS-DA models consisting in 500 random permutations. (A), (B), (C) Validation plots of the PLS-DA models of t_45H_, t_45H_ after exercise and t_+21N_ after exercise, respectively. Table S1: Effects of mild-hypoxia conditioning and the subsequent normoxia recovery (1 or 3 weeks thereafter) on plasma and muscle tissue levels of metabolites and hormones after exercise exhaustion of gilthead sea bream juveniles. Values are the mean ± SEM of 6–7 fish (2–3 fish per replicate tank). *p*-values are the result of one-way ANOVA or *t*-test analysis where appropriate. Different superscript letters or asterisks (*) indicate significant differences between experimental groups (*p* < 0.05). Table S2: Statistics of the preprocessing and mapping of RNA-seq libraries. t_45H_, t_45H_-Ex and t_+21N_ correspond to experimental groups conditioning, Exercise in t_45H_ and Exercise in t_+21N_, respectively. N, N-PF and M-HYP correspond to the different fish conditions: Normoxia, normoxia pair-fed and mild-hypoxia, respectively. Table S3: Differentially expressed genes and associated enriched pathways.
