# Supplementary material for: Targeting the Mild-Hypoxia Driving Force for Metabolic and Muscle Transcriptional Reprogramming of Gilthead Sea Bream (Sparus aurata) Juveniles
Source: Biology (Basel). 2021 May 8;10(5):416. doi: 10.3390/biology10050416 (PMC8151949; doi:10.3390/biology10050416)
Supplement: Supplementary file 1 [file biology-10-00416-s001.zip › Table S1. Blood biochemestry exhaustion.pdf]

Table S1. Effects of mild hypoxia conditioning and the subsequent normoxia recovery (1 or 3 weeks thereafter) on plasma and muscle tissue levels of metabolites and hormones after exercise exhaustion of gilthead sea bream juveniles. Values are the mean ± SEM of 6-7 fish (2-3 fish per replicate tank). P-values are the result of one-way ANOVA or *t*-test analysis where appropriate. Different superscript letters or asterisks (*) indicate significant differences between experimental groups (P <0.05).

	N	N-PF	M-HYP	P-value
Sampling time: T_{45H}				
Glucose (mg/dl)	215.8 ± 12.3	158.9 ± 23.9	179.9 ± 28.1	0.227
Lactate (mg/dl)	103.7 ± 4.41^{b}	53.3 ± 3.12^{a}	92.3 ± 5.06^{b}	<0.001
Muscular lactate (mg/g bm)	50.9 ± 1.93^{b}	44.9 ± 0.75^{a}	56.1 ± 1.56^{b}	<0.001
Triglycerides (mg/dl)	2.99 ± 0.49	2.35 ± 0.54	1.82 ± 0.15	0.187
Free fatty acids (nmol/µl)	0.490 ± 0.06^{a}	1.00 ± 0.30^{ab}	1.14 ± 0.32^{b}	0.027
Cortisol (ng/ml)	235.0 ± 23.7^{b}	88.2 ± 33.84^{a}	160.3 ± 41.65^{ab}	0.026
Growth hormone (ng/ml)	16.2 ± 7.13	9.36 ± 4.15	9.02 ± 2.28	0.529
Insulin-like growth factor-I (ng/ml)	77.3 ± 5.65^{b}	58.6 ± 4.25^{a}	61.5 ± 4.76^{a}	0.035
Sampling time: T_{+7N}				
Glucose (mg/dl)	225.6 ± 10.2		168.6 ± 17.4*	0.018
Lactate (mg/dl)	104.1 ± 6.57		124.1 ± 2.64*	0.017
Muscular lactate (mg/g bm)	50.6 ± 1.86		59.3 ± 3.13*	0.039
Triglycerides (mg/dl)	2.51 ± 0.55		1.88 ± 0.24	0.323
Free fatty acids (nmol/µl)	0.423 ± 0.031		0.789 ± 0.301	0.253
Cortisol (ng/ml)	293.5 ± 41.2		165.3 ± 46.6	0.066
Growth hormone (ng/ml)	21.8 ± 3.51		10.5 ± 2.84*	0.031
Insulin-like growth factor-I (ng/ml)	77.9 ± 4.70		65.7 ± 7.09	0.102
Sampling time: T_{+21N}				
Glucose (mg/dl)	211.9 ± 11.8		210.5 ± 13.5	0.936
Lactate (mg/dl)	104.5 ± 7.2		126.4 ± 4.6*	0.025
Muscular lactate (mg/g bm)	51.5± 2.03		59.0 ± 1.72*	0.015
Triglycerides (mg/dl)	2.7 ± 0.32		2.75 ± 0.25	0.836
Free fatty acids (nmol/µl)	0.54 ± 0.119		0.635 ± 0.114	0.549
Cortisol (ng/ml)	235.2 ± 44.6		246.7 ± 19.4	0.818
Growth hormone (ng/ml)	11.13 ± 4.8		12.0 ± 2.43	0.874
Insulin-like growth factor-I (ng/ml)	72.2 ± 6.14		72.0 ± 6.20	0.983
