# Supplementary material for: Targeting the Mild-Hypoxia Driving Force for Metabolic and Muscle Transcriptional Reprogramming of Gilthead Sea Bream (Sparus aurata) Juveniles
Source: Biology (Basel). 2021 May 8;10(5):416. doi: 10.3390/biology10050416 (PMC8151949; doi:10.3390/biology10050416)
Supplement: Supplementary file 1 [file biology-10-00416-s001.zip › Table S2. Sequencing_Data.pdf]

Table S2. Statistics of the mapping of RNA-seq libraries.

Group	Raw reads	Total clean reads	Total mapped reads	Unique mapped reads	Multiple mapped reads	Unmapped reads
T_{+45H}-N (x6)	93,962,710	91,112,977 (100%)	75,992,423 (83.40%)	56,420,759 (61.92%)	19,571,664 (21.48%)	15,120,554 (16.60%)
T_{+45H} -N-PF (x6)	106,280,617	103,264,916 (100%)	85,542,148 (82.84%)	62,918,817 (60.93%)	22,623,331 (21.91%)	17,722,768 (17.16%)
T_{+45H} -M-HYP (x6)	111,987,385	109,287,035 (100%)	90,248,445 (82.58%)	66,082,408 (60.47%)	24,166,037 (22.11%)	19,038,590 (17.42%)
T_{+45H} -Ex-N (x6)	113,249,761	109,802,281 (100%)	90,724,845 (82.63%)	67,297,236 (61.29%)	23,427,609 (21.34%)	19,077,436 (17.37%)
T_{+45H} -Ex-N-PF (x6)	106,192,018	103,642,199 (100%)	86,316,417 (83.28%)	63,380,774 (61.15%)	22,935,643 (22.13%)	17,325,782 (16.72%)
T_{+45H}-Ex-M-HYP (x6)	104,507,973	101,731,147 (100%)	84,192,210 (82.76%)	62,282,546 (61.22%)	21,909,664 (21.54%)	17,538,937 (17.24%)
T_{+21N} -N (x7)	122,629,685	119,165,470 (100%)	98,043,378 (82.27%)	72,890,156 (61.17%)	25,153,222 (21.11%)	21,122,092 (17.73%)
T_{+21N} -M-HYP (x7)	125,560,548	121,690,996 (100%)	100,665,832 (82.72%)	74,463,326 (61.19%)	26,202,506 (21.53%)	21,025,164 (17.28%)

T_{+45H}, T_{+45H}-Ex and T_{+21N} correspond to experimental groups conditioning, Exercise in T_{+45H} and Exercise in T_{+21N}, respectively. N, N-PF and M-HYP correspond to the different fish conditions: Normoxia, normoxia pair-feed and mild-hypoxia, respectively.
