# Supplementary figures and images for: Targeting the Mild-Hypoxia Driving Force for Metabolic and Muscle Transcriptional Reprogramming of Gilthead Sea Bream (Sparus aurata) Juveniles
Source: Biology (Basel). 2021 May 8;10(5):416. doi: 10.3390/biology10050416 (PMC8151949; doi:10.3390/biology10050416)

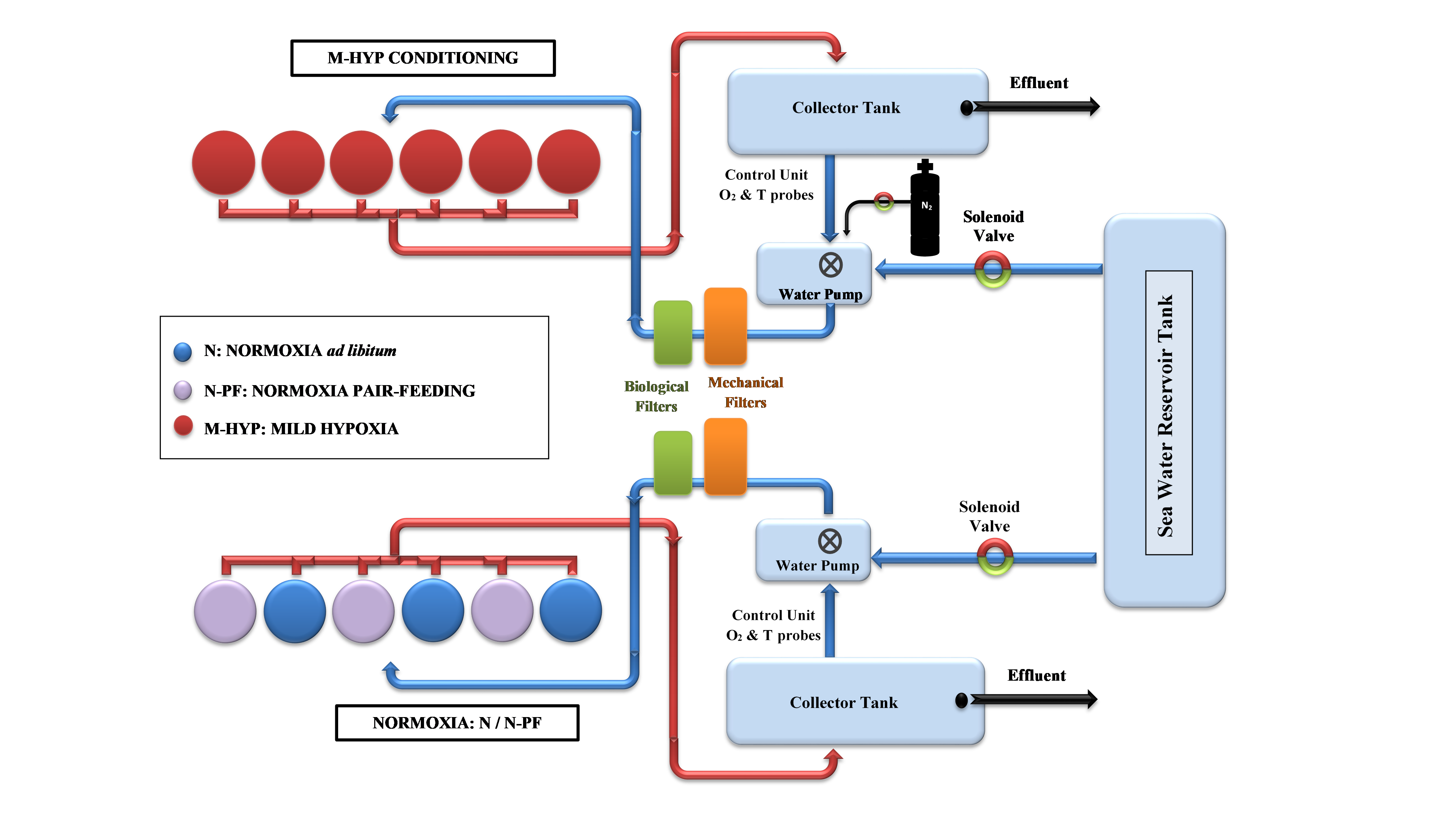

Supplement: Supplementary file 1 [file biology-10-00416-s001.zip › Figure S1. Closed System Diagram.tif]

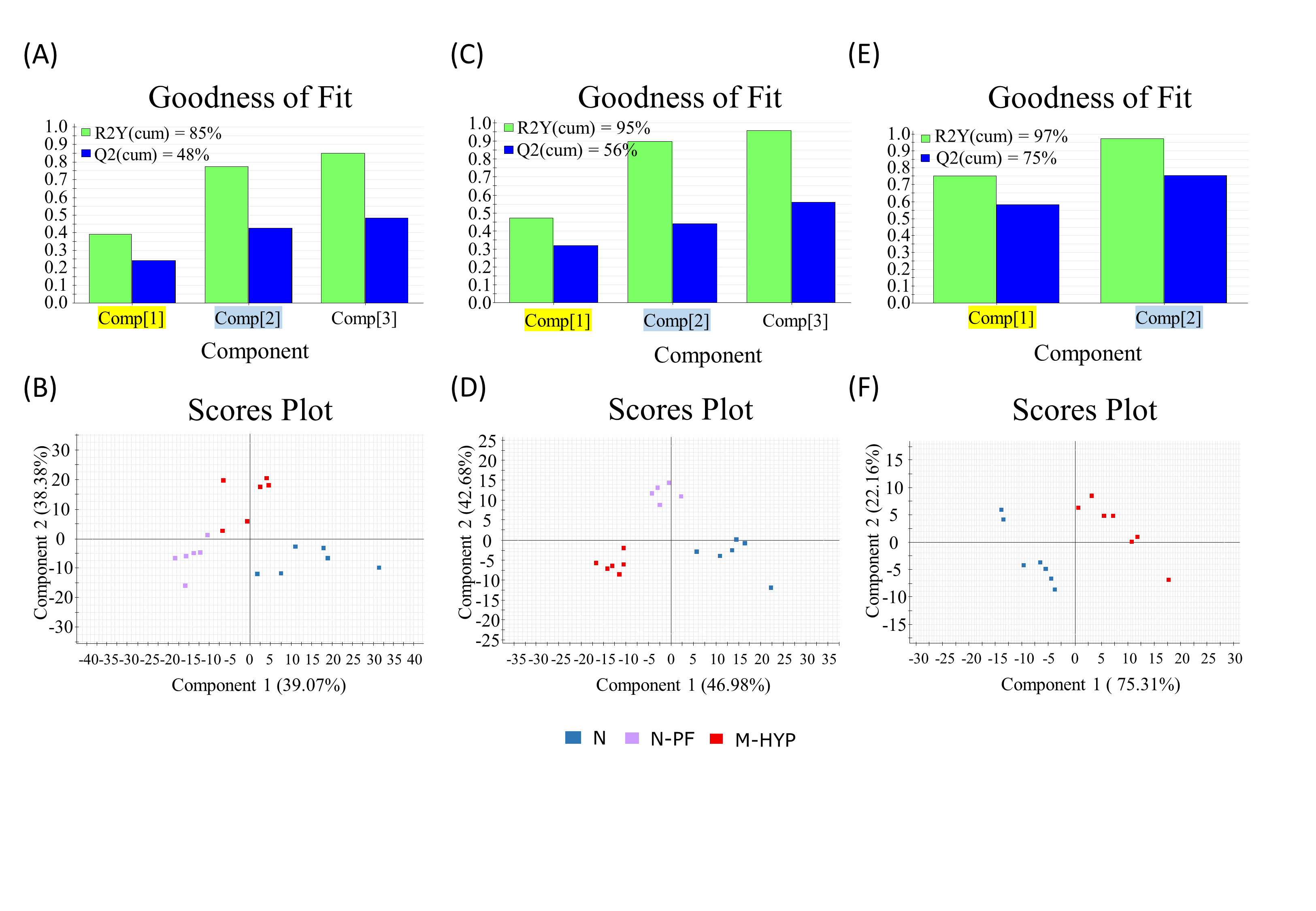

Supplement: Supplementary file 1 [file biology-10-00416-s001.zip › Figure S2 PLS-DA.tif]

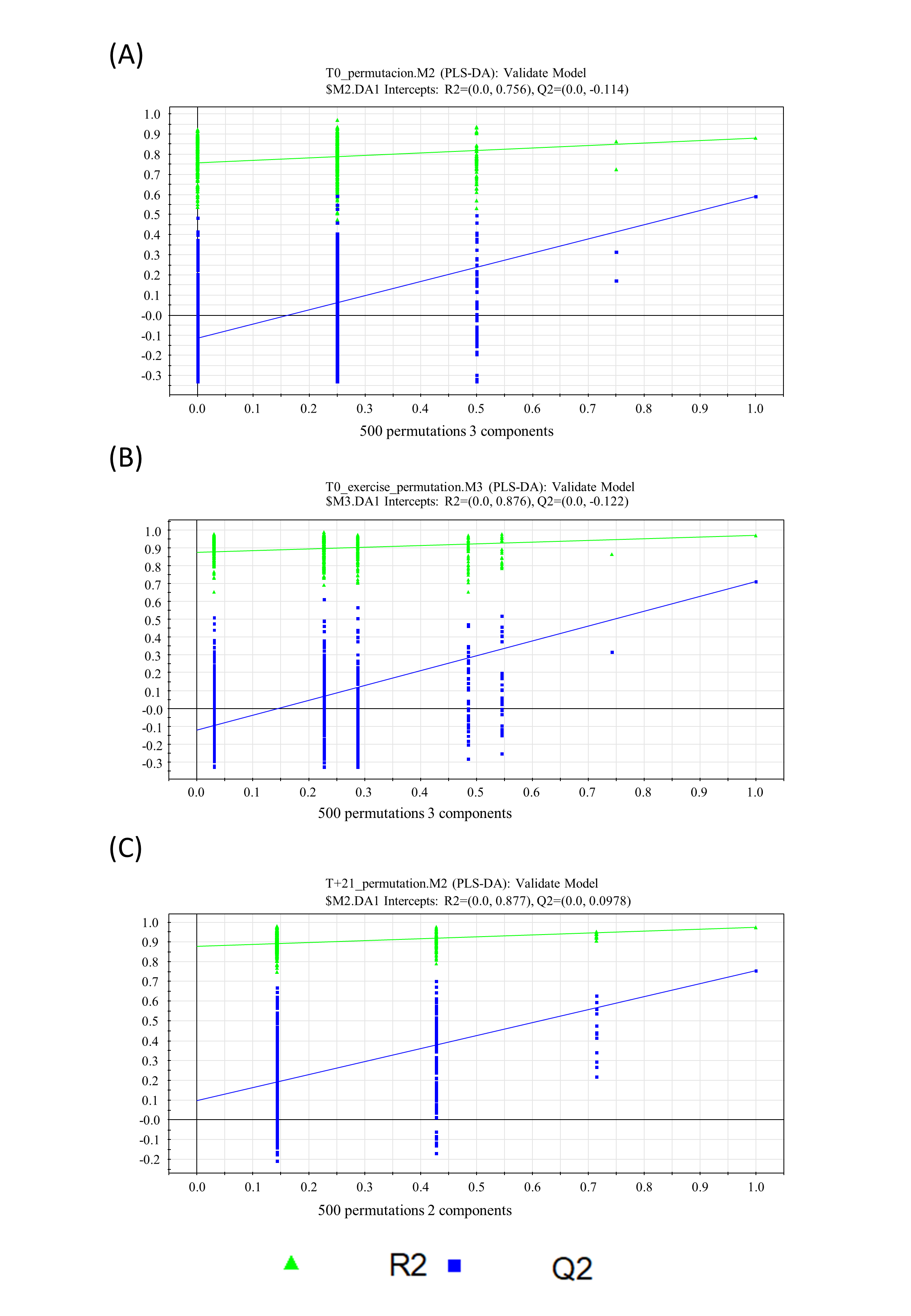

Supplement: Supplementary file 1 [file biology-10-00416-s001.zip › Figure S3. Permutation_plots_pls-da.tif]
